# Small Cell Type Poorly Differentiated Neuroendocrine Cancer of the Gallbladder

**DOI:** 10.7759/cureus.85733

**Published:** 2025-06-10

**Authors:** Akansha, Madhumita Tripathi, Roli Purwar, Mridula Shukla, Manoj Pandey

**Affiliations:** 1 Surgery, Institute of Medical Sciences, Banaras Hindu University, Varanasi, IND; 2 Surgical Oncology, Institute of Medical Sciences, Banaras Hindu University, Varanasi, IND; 3 Pathology, Dr. Lalpath Labs Pvt. Ltd, Varanasi, IND

**Keywords:** gallbladder, hepatobiliary cancer, neuroendocrine cancer, neuroendocrine tumor (net), small cell carcinoma

## Abstract

Neuroendocrine tumor (NET) is a rare gallbladder (GB) malignancy and is seldom seen in clinical practice. There is a scarcity of reported cases, or extensive studies, hence not much is known about the disease. We present here the case of a 62-year-old woman, presenting with jaundice as the only symptom. On subsequent investigations, it was diagnosed to be poorly differentiated neuroendocrine carcinoma (NEC) of the GB with invasion of adjoining organs and peritoneal metastasis and was managed with palliative chemotherapy. At present, the treatment of choice for GB NET is surgical resection, but it is possible in only very limited cases due to metastatic disease at the time of first presentation. Hence, in such cases, chemotherapy remains a feasible alternative.

## Introduction

Neuroendocrine tumors (NETs) are a heterogeneous group of tumors arising from neuroendocrine cells. Most of the data on NET comes from Western literature, with data on Indian patients lacking. The average incidence of gastrointestinal (GI) NETs is 2.5 cases per 100,000 per year in the United States [1]. According to the data from the Surveillance, Epidemiology and End Result (SEER) program, gallbladder (GB) NET accounts for 0.5% of all NET and 2.1% of all GB tumors; the incidence has been shown to be on the rise and could be due to increased detection rates [2].

NETs can arise from any part of the body; however, the GI and pancreaticobiliary tracts are common sites [3]. These can have a varied clinical picture which primarily depends on tumor biology, histology, and their secretary nature if any [4]. It is difficult to differentiate NET from adenocarcinoma based on the imaging, and hence, each case is to be treated differently depending upon their inherent variations. These are very aggressive tumors with poor survival outcomes despite treatment. Here we report a case of small cell NET presenting with obstructive jaundice and a review of the literature.

## Case presentation

In February 2020, a 62-year-old woman, a housewife by occupation, presented to the surgical oncology outpatient with complaints of abdominal pain localized to the right upper quadrant along with yellowish discoloration of sclera for the last one month. She had no known comorbidities and gave no past history of any surgical interventions. She had undergone endoscopic retrograde cholangiopancreatography (ERCP) with placement of a 10 x 10 Fr straight plastic stent in the common bile duct (CBD), for the control of jaundice and its related symptoms one week prior to surgical oncological consultation. On ERCP a long stricture was found in mid to upper CBD with carcinoma of the GB.

On abdomen examination, a 7 x 9 cm hard mass with a smooth surface was palpated in the right hypochondrium extending into the right lumbar and umbilical region whose upper margin couldn’t be made out, but the rest of the mass was well appreciated. The mass was mobile in the perpendicular axis and showed movement with respiration. Rectal and vaginal examinations were normal with no palpable growth. Bilateral supraclavicular fossae were free. The patient was found to be icteric, other hematological and biochemical parameters were within normal range. ECOG (Eastern Cooperative Oncology Group) status was 2.

**Figure 1:**
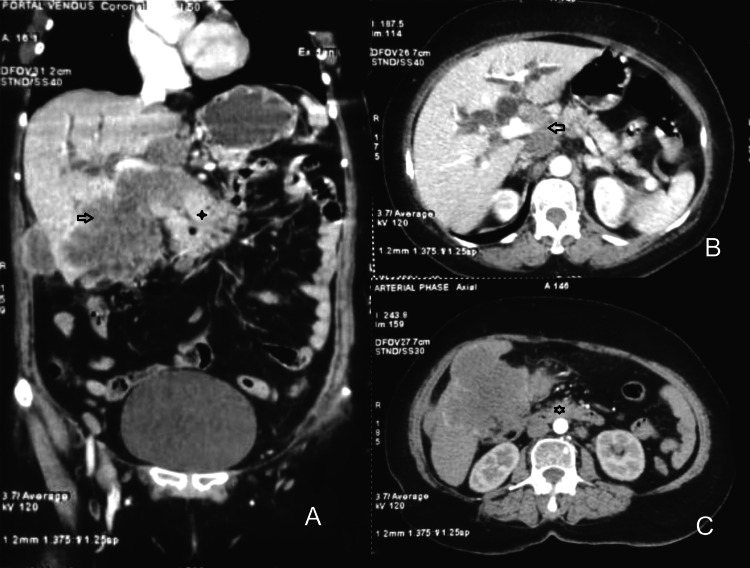
CT scan panel showing Ill-defined heterogeneous, peripherally enhancing mass lesion, with internal non-enhancing necrotic areas having exophytic component, arising from gall bladder involving segment IVb & V of the liver (arrow), medially involving antropyloric region of the stomach (plus marker), and inferiorly infiltrating hepatic flexure of the colon, also infiltrating overlying muscular layer of the anterior abdominal wall (A). The lesion is encasing the portal vein causing irregular luminal narrowing along with IHBRD due to infiltration of the common bile duct (B). Enlarged retroperitoneal lymph node noted (star marker) (C). IHBRD: intrahepatic biliary radicle dilatation

She underwent a triphasic CT scan of the abdomen which showed an ill-defined heterogeneously enhancing soft tissue density mass lesion of size 9 x 9 x 10 cm, in gallbladder fossa. The lesion infiltrated the adjacent hepatic parenchyma (segments IVb and V), the antropyloric region of the stomach, the second part of the duodenum (causing significant luminal narrowing), the hepatic flexure of the colon, and the common hepatic and common bile ducts along their entire course up to the biliary confluence, which remained patent. There were multiple enlarged, necrotic intrabdominal lymph nodes along with lateral abdominal wall deposits (Figure 1A-1C). Her tumor antigens, CEA, and CA 19-9 were 0.07 ng/ml and 0.024 U/ml respectively, both being within the normal range.

**Figure 2:**
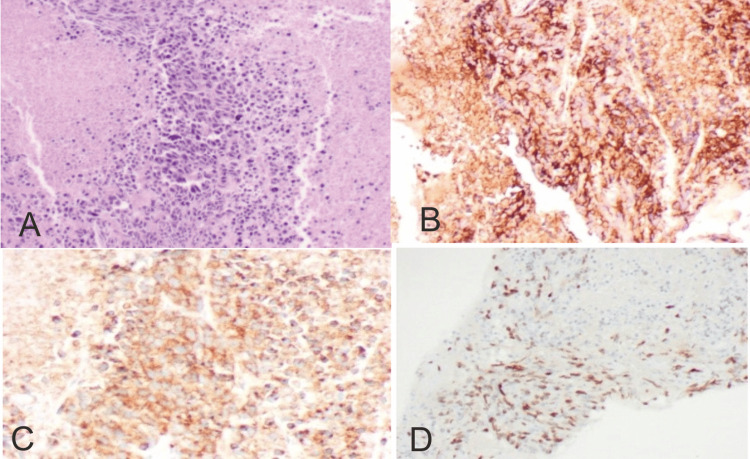
Photomicrograph revealing a high-resolution image of poorly differentiated carcinoma, suggestive of small cell neuroendocrine carcinoma (A). Immunohistochemistry (IHC) demonstrates: CD56, immunoreactive score 2+ (26–50%) in neoplastic cells (B); synaptophysin, immunoreactive score 3+ (51–75%) in neoplastic cells (C); and Ki-67, staining 40% of tumor cells (D).

Further evaluating the patient, an ultrasonography-guided biopsy was performed for the GB mass that on histopathology examination was found to be a poorly differentiated carcinoma-favoring small cell neuroendocrine tumor. On immunohistochemistry, cells were strongly positive for synaptophysin and CD56 & Ki67 were 40%, which confirmed the diagnosis of small cell neuroendocrine tumor of the GB (Figure 2A-2D).

A multidisciplinary team was consulted, and the patient was started on paclitaxel and carboplatin-based palliative chemotherapy keeping in mind her metastatic disease status. Assessment of response to chemotherapy could not be achieved, as after receiving two cycles of chemotherapy she succumbed to death due to community-acquired atypical pneumonia leading to cardiorespiratory arrest. Her COVID-19 RT-PCR (reverse transcription-polymerase chain reaction) was negative and her blood culture and bronchial lavage were negative for bacterial growth. Viral pneumonitis was suspected as the cause of death.

## Discussion

Neuroendocrine tumors (NETs) are epithelial neoplasms with predominant neuroendocrine differentiation arising from neuroendocrine cells. They can arise in most organs and are widely distributed throughout the body; however, their occurrence in the GB is rare [3-5].

The results from SEER data showed the lung as the most common site of primary NET, whereas other studies showed the small intestine and rectum to be common primary sites [1]. In Indian studies stomach was the most common site (30.2%), followed by the pancreas (22.3%) [5-9]. Other Indian studies reported the pancreas to be the most common primary site seen [6-9].

NETs including those arising from GI and pancreatobiliary tracts are classified as per the WHO 2022 classification based on the degree of differentiation into well-differentiated, and poorly differentiated endocrine tumors [10]. Based on the proliferative rate (mitotic rate and Ki-67 index) the well-differentiated NETs are further subdivided into a low grade (G1) and intermediate grade (G2) while grade 3 are classified as poorly differentiated endocrine carcinomas (G3) while the small cell and large cell carcinomas are classified as A type of neuroendocrine carcinoma (NEC) under poorly differentiated cancers [10]. Based on their ability to produce peptides, NETs are further classified as being functional and nonfunctional.

Biliary tract NETs are extremely rare, comprising 0.2% of all GI carcinoids and small cell NET of the GB is even rarer with only about 100 cases reported in the literature. However, there is no consensus on their presentation that varied from case to case and on their management. As the GB does not have neuroectodermal cells, it is proposed that the primary NET hypothetically arises from a pluripotent stem cell or metaplasia of the gallbladder mucosa as a result of chronic insult caused by the bacteria or gallstones, as most cases are associated with gallstones. However, cases without association with gallstones too are reported.

The varied presentation of this tumor is due to its location within the gallbladder and histological type and this includes mass effect, pain, bleeding, jaundice, or the classic carcinoid syndrome. The majority of NETs in the gallbladder are non-functional APUD (amine precursor uptake and decarboxylation) tumors and hence are often diagnosed late when the disease is beyond a surgical cure [11].

NET progresses rapidly and induces early liver invasion and lymphatic and peritoneal metastasis. Due to the lack of systemic symptoms, it often presents at an advanced stage and is difficult to differentiate from adenocarcinoma of the gallbladder which is very common in India and share the same presenting symptoms and imaging findings. In about 10% of the cases, the disease is surgically amicable while in the rest a needle biopsy followed by chemotherapy or radiotherapy is used. In a report of 37 cases from China, only 21 underwent surgical resections [12].

Due to the rarity of this neoplasm, only a few studies on the radiology of these tumors are available and authors have tried to differentiate it from adenocarcinoma on ultrasound (USG) [13], computed tomography (CT) [14], or magnetic resonance imaging (MRI) [15]; however, no characteristic findings are identified. In our patient, the CT showed a large, infiltrating mass, while the laboratory workup including CEA and CA 19-9 was unremarkable. Histopathology is the only way to definitely diagnose and characterize these tumors.

Complete surgical resection with negative margins is the best treatment option available at present. In the literature, they have varied from radical cholecystectomy to simple cholecystectomy by the open or laparoscopic route. However, even this is possible only in 10% of patients. The use of adjuvant or neoadjuvant chemotherapy with surgical resection is shown to increase survival from 4.5 to 13 months [16,17].

Chemotherapy plays a limited role in non-pancreatic NETs and has shown some benefit, although the optimal regimen remains unclear. Based on the literature, the most commonly used chemotherapy drugs include streptozotocin, 5-fluorouracil, adriamycin, cisplatin, carboplatin, and etoposide [17,18]. Despite the report of over 100 cases, there is still a lack of data on effective chemotherapy regimens and the use of concurrent chemoradiation. Literature shows oxaliplatin and gemcitabine to be the most used chemotherapeutic agents with poor response rates and it appears that these regimens have been used thinking it to be an adenocarcinoma without histological confirmation and hence poor response rates. Immunotherapy with nivolumab and ipilimumab has been tried in just one case with a durable response [19]. The chemotherapy drugs advocated for NEC include etoposide, cisplatin, and Adriamycin. Attempts have been made on molecular characterization and beside mutations of TP53, RAD54L, ATM, and BRCA2; amplifications of ERBB2 and MDM2; and a gene fusion involving FGFR3-TACC3 has also been reported [20].

## Conclusions

Neuroendocrine tumor of gallbladder is a rare tumor with very few cases reported worldwide, hence there is a lack of guidelines in its diagnosis and treatment. The literature suggests that the early presentation of this cancer can be managed through surgery, but there is still no approved treatment for advanced diseases apart from chemotherapy, whose usefulness cannot be ascertained. Hence, one need to be vigilant of its presence and try for an as early an identification as possible, though difficult as it mimics the adenocarcinoma of the gallbladder in its presentation and imaging findings and diagnosis is always made after a biopsy and immunohistochemistry.
